# What Man Is Made of

**Published:** 1890-07

**Authors:** 


### WHAT MAN IS MADE OF.

Dr. Lancaster, a London physician and surgeon, recently analyzed a man, and gave the results to his class in chemistry. The body operated upon weighed 154.4 pounds. The lecturer exhibited upon the platform 23.1 pounds of carbon, 2.2 pounds of lime, 22.3 ounces phosphorus and about one ounce each of sodium, iron, potassium, magnesium and silicon. Besides this solid residue, Dr. Lancaster estimated that there were 5,595 cubic feet of oxygen, weighing 121 pounds; 105,900 cubic feet of hydrogen, weighing 15.4 pounds, and 52 cubic feet of nitrogen in the man's body. All of these elements combined in the following: One hundred and twenty-one pounds of water. 16.5 pounds gelatine, 1.32 pounds fat, 8.8 pounds fibrin and albumen, and 7.7 pounds of posphate of lime and other minerals.

